# Genome-wide association data suggest *ABCB1* and immune-related gene sets may be involved in adult antisocial behavior

**DOI:** 10.1038/tp.2015.36

**Published:** 2015-04-28

**Authors:** J E Salvatore, A C Edwards, J N McClintick, T B Bigdeli, A Adkins, F Aliev, H J Edenberg, T Foroud, V Hesselbrock, J Kramer, J I Nurnberger, M Schuckit, J A Tischfield, X Xuei, D M Dick

**Affiliations:** 1 Virginia Institute for Psychiatric and Behavioral Genetics, Department of Psychiatry, Virginia Commonwealth University, Richmond, VA, USA; 2 Department of Biochemistry and Molecular Biology, Indiana University, Indianapolis, IN, USA; 3 Department of Statistics and Institute of Biotechnology, Ankara University, Ankara, Turkey; 4 Department of Medical and Molecular Genetics, Indiana University, Indianapolis, IN, USA; 5 Department of Psychiatry, University of Connecticut, Farmington, CT, USA; 6 Department of Psychiatry, University of Iowa, Iowa City, IA, USA; 7 Department of Psychiatry, Indiana University, Indianapolis, IN, USA; 8 Department of Psychiatry, University of California San Diego, La Jolla, CA, USA; 9 Department of Genetics, Rutgers University, Piscataway, NJ, USA

## Abstract

Adult antisocial behavior (AAB) is moderately heritable, relatively common and has adverse consequences for individuals and society. We examined the molecular genetic basis of AAB in 1379 participants from a case–control study in which the cases met criteria for alcohol dependence. We also examined whether genes of interest were expressed in human brain. AAB was measured using a count of the number of Antisocial Personality Disorder criteria endorsed under criterion A from the Diagnostic and Statistical Manual of Mental Disorders, 4th Edition (DSM-IV). Participants were genotyped on the Illumina Human 1M BeadChip. In total, all single-nucleotide polymorphisms (SNPs) accounted for 25% of the variance in AAB, although this estimate was not significant (*P*=0.09). Enrichment tests indicated that more significantly associated genes were over-represented in seven gene sets, and most were immune related. Our most highly associated SNP (rs4728702, *P*=5.77 × 10^−7^) was located in the protein-coding adenosine triphosphate-binding cassette, sub-family B (MDR/TAP), member 1 (*ABCB1*). In a gene-based test, *ABCB1* was genome-wide significant (*q*=0.03). Expression analyses indicated that *ABCB1* was robustly expressed in the brain. *ABCB1* has been implicated in substance use, and in *post hoc* tests we found that variation in *ABCB1* was associated with DSM-IV alcohol and cocaine dependence criterion counts. These results suggest that *ABCB1* may confer risk across externalizing behaviors, and are consistent with previous suggestions that immune pathways are associated with externalizing behaviors. The results should be tempered by the fact that we did not replicate the associations for *ABCB1* or the gene sets in a less-affected independent sample.

## Introduction

Adult antisocial behavior (AAB), which is broadly defined as a ‘pervasive pattern of disregard for and violation of the rights of others',^1^ is relatively common and is associated with adverse consequences for both individuals and societies. According to prevalence estimates from the 2001–2002 National Epidemiologic Survey on Alcohol and Related Conditions, 3.63% (95% confidence interval=3.34–3.92) of adult Americans qualify for an antisocial personality disorder (ASPD) diagnosis, and men are affected at a greater rate than women.^2^ Findings from the all-male Vietnam Era Twin Registry indicate that ASPD is highly heritable, with genetic factors accounting for 69% of the variation in diagnoses.^3^ Twin and adoption studies further indicate that dimensional measures of AAB are moderately heritable, with genetic factors accounting for ~40% of the variation in these behaviors.^4, 5, 6^ These figures are similar to the results from a meta-analysis of 51 twin and adoption studies from child, adolescent and adult samples, which found that additive genetic influences accounted for 32% of the variance in antisocial behavior.^7^

A number of studies of AAB have found statistically significant genetic associations or gene-by-environment interactions with measures of early environmental adversity for variants in candidate genes or gene regions (for example, *5-HTTLPR*, *COMT* and *MAOA*).^8, 9, 10^ A recent meta-analysis found evidence for associations between antisocial behavior (measured across a range of ages) and variation in the serotonin transporter gene *SLC6A4* (that is, the *5-HTTLPR* polymorphism) and the monoamine oxidase A (*MAOA*) gene.^11^ Although there has been longstanding controversy surrounding failures to replicate and publication bias for candidate gene approaches,^12^ it was only recently that researchers began to systematically examine how common genetic variation across the genome is associated with AAB. No single-nucleotide polymorphisms (SNPs) met genome-wide significance in the genome-wide association study (GWAS) by Tielbeek *et al.*^13^ of non-diagnostic categorical measures of AAB in two Australian samples. The most highly associated gene to emerge from their analysis was *DYRK1A* (*P=*8.7 × 10^−5^) on chromosome 21, which is a candidate gene for developmental disabilities.^14^ In this same study, attempts to replicate the associations between seven candidate genes or polymorphisms from the literature (*DAT1*, *DRD2*, *DRD4*, *5-HTTLPR*, *COMT*, *MAOA* and *C1QTNF7*) and AAB as a categorical variable were unsuccessful. Likewise, studies in a community-based sample reported no significant associations between a closely related behavioral disinhibition phenotype and rare nonsynonymous exonic or common SNPs from across the genome.^15, 16^

Additional screens of high-risk samples that are likely to be enriched for AAB are needed to detect genetic associations. Our goal in the present study was to fill this gap and examine the molecular genetic basis of AAB in such a sample. We examined SNP heritability of a dimensional measure of AAB, followed by a GWAS, gene-based tests and gene set enrichment tests. Participants were part of a case–control sample in which cases met the criteria for alcohol dependence. Alcohol-use disorders are phenotypically and genetically correlated with AAB and ASPD,^17, 18^ suggesting that this sample is likely to be enriched for variants contributing to AAB. We then examined whether genes of interest were expressed in human brain to evaluate the biological plausibility that identified genes would be associated with a behavioral outcome.

## Materials and methods

### Sample and participants

Participants came from the Collaborative Study on the Genetics of Alcoholism (COGA).^19^ The primary goal of COGA is to identify genes involved in alcohol dependence and related disorders. Alcohol-dependent probands were identified through alcohol treatment programs at seven US sites and were invited to participate if they had a sufficiently large family (usually sibships >3 with parents available) with two or more members in the COGA catchment area. Community probands and their families were recruited through driver's license records, mailings to randomly selected employees and students at a university, and attendees at medical and dental clinics. The institutional review boards at all sites approved this study and written consent was obtained from all participants. The present study included the participants in the European-American subset from the COGA case–control GWAS sample (in which cases met Diagnostic and Statistical Manual of Mental Disorders, 4th Edition (DSM-IV) alcohol dependence criteria^20^) for whom adult antisocial behavior interview data were also available (*n*=1379; 739 (54%) male and 640 (46%) female). European ancestry was determined using a principal-component-based analysis in PLINK.^20^ The average age at assessment was 43.8 years (s.d.=11.7; range=18–79).

### AAB

AAB was measured using a count of the number of ASPD criteria endorsed under criterion A from the DSM-IV.^1^ In view of our modest sample size, we chose to use a more powerful dimensional phenotype rather than a less powerful diagnostic phenotype. The seven criteria included: engaging in illegal activities; deceitfulness; impulsivity and failing to plan ahead; irritability and aggressiveness; disregard for the safety of self and others; consistent irresponsibility at work or with finances; and lack of remorse. Criterion counts were obtained from items in the reliable (with a kappa=0.70 for ASPD diagnoses) and valid Semi-Structured Assessment for the Genetics of Alcoholism (SSAGA).^21, 22^ We note that 203 (15%) of participants were administered an early version of the SSAGA that included a skip pattern whereby participants were asked the ASPD questions only if they had endorsed two or more alcohol dependence/abuse, marijuana dependence/abuse or other drug dependence/abuse symptoms. In total, 42 participants (representing 3% of the overall sample) were not asked the ASPD questions because of the skip pattern.

### Genotyping and imputation

Participants were genotyped on the Illumina Human 1M BeadChip (San Diego, CA, USA); detailed genotyping and quality control information for this sample can be found in Edenberg *et al.*^20^ The European-American and African-American participants in the case–control sample were imputed together (*n*=1884). Prior to imputation, monomorphic SNPs (that is, SNPs with a minor allele frequency <0.0001), SNPs with missingness >2%, SNPs with Hardy–Weinberg equilibrium *P*<1 × 10^−6^ and SNPs that did not map to the Hg19 reference genome were filtered out, leaving 936 240 SNPs. Phasing of entire chromosomes or chromosome arms was performed using SHAPEIT.^23, 24^ Genotype imputation of additional SNPs was carried out with IMPUTE2 v.2.031 using the March 2012 release (v3) of the 1000 Genomes Project data (www.1000genomes.org^25^). Imputation analysis was performed for genomic windows of 5 Mb with an overlap interval of 500 kb between adjacent segments. Following the recommendations of the authors of IMPUTE2, we did not limit our imputation procedure to European reference samples. Monomorphic sites were excluded.

### Analytic plan

We used genome-wide complex trait analysis (GCTA^26^) to estimate the extent to which autosomal common genetic variation accounted for variance in AAB. GCTA estimates the genetic relationships among individuals in a sample from genome-wide SNP data, and then uses a mixed-linear modeling framework to estimate heritability. The Illumina 1M BeadChip provides sufficient coverage of variation across the genome for the purposes of estimating common SNP heritability using GCTA; accordingly, we used non-imputed genotypic data for this analysis.

For the GWAS, the imputed gene dosage data were analyzed in PLINK.^27^ Covariates included: cohort (a four-category variable based on year of birth that was included to control for potential cohort effects: 1896–1929; 1930–1949; 1950–1969; and after 1970), age at assessment, sex and the first four principal components derived from a principal component-based analysis performed in PLINK on the measured genotypic data to cluster the samples along with HapMap reference samples (CEU, YRI, CHB and JPT). Results were filtered to include only SNPs with minor allele frequency >0.01 that also met an information criterion of >0.80. In total, 7 287 851 SNPs met these criteria.

We used KGG 2.5 (refs. 28, 29) to conduct gene-based tests of our GWAS results. This gene-based test examines whether the set of SNPs in a gene is associated with the phenotype at a level greater than chance. We used publicly available 1000 Genomes Phase 1 version 3 (European subsample) linkage disequilibrium (LD) files to build the ‘analysis genome' by position, for autosomes only. We included extended gene lengths of 5 kb at both the 5′ and 3′ ends. SNPs in high LD (*r*^2^>0.9) were connected; those in low LD (*r*^2^ <0.02) were considered independent. We used the hybrid set-based test for genome-wide association studies^29^ and report both the uncorrected *P*-values as well as the *q*-value based on a Benjamini and Hochberg^30^ false discovery rate. *q*-values <0.05 are considered genome-wide significant. Following this, we used *i*-GSEA4GWAS^31^ to assess enrichment across canonical pathways (defined by the authors of the *i*-GSEA4GWAS program) and gene ontologies. In total, 23 153 genes were submitted along with *P*-values (which were log-transformed by *i*-GSEA4GWAS). We set the minimum number of genes per category to 20, and the maximum to 200.

Genes of interest (that is, meeting a *P*-value threshold of less than 1 × 10^−4^) were compared with microarray data from nine brain regions (prefrontal cortex, cerebral cortex, thalamus, visual cortex, hippocampus, amygdala, caudate nucleus, putamen and cerebellum) from post-mortem tissue from two males and two females, which included an alcoholic and a control of each sex, to determine whether they were expressed in the brain.^32^ These nine regions were chosen to provide broad coverage across the brain. The expression values for our genes of interest did not substantially vary across these regions, and we therefore report the maximum of the average expression value. A gene was presumed to be expressed in the brain if the maximum expression level across the nine regions was higher than 16.0, because this is above the background signal for the arrays.

### Robustness check and pleiotropy analyses

The sample used in the present study was originally recruited as part of a case–control GWAS of alcohol dependence. To check that our results were not simply driven by the phenotypic association between AAB and alcohol dependence, we examined whether the genome-wide significant gene to emerge from our analysis of AAB remained at least nominally significant after statistically controlling for DSM-IV alcohol dependence case–control status using a gene-based test. We also conducted a series of *post hoc* gene-based pleiotropy analyses to examine whether the genome-wide significant gene to emerge from our analysis showed evidence for association across a broader range of genetically correlated externalizing phenotypes.^18^ To pursue this, we examined association between our gene of interest and DSM-IV criterion counts for four major drug classes: alcohol dependence, cannabis dependence, cocaine dependence and opioid dependence. Because these were separate analyses of correlated phenotypes, a Bonferroni correction would be too stringent. We therefore used a nominal *P*-value cutoff of *P*<0.05 as evidence for association.

### Replication study

Independent replication was tested in the COGA family-based GWAS (fGWAS) sample. The fGWAS sample includes 118 European-American COGA families densely affected with alcohol dependence (at least 3+ affected members) for whom genome-wide association data are available.^33^ The 270 individuals from the COGA case–control sample who were also in the fGWAS sample were removed from the replication data set. In total, AAB data from 1796 individuals in the fGWAS sample were available for analysis. Of these, 823 (46%) participants were male and 973 (54%) were female. The average age at assessment was 36.2 years (s.d.=15.2; range=18–88). AAB was measured using a count of the number of endorsed ASPD criteria under criterion A from the DSM-IV, as for our initial analysis. Association analyses in this sample were run using GWAF,^34^ which accounts for familial nesting and genetic distance using a kinship matrix. Covariates included cohort, age at assessment, sex and the first four principal components to control for genetic ancestry. Unimputed genotypic data were used.

## Results

In the case–control sample, participants endorsed an average of 2.56 AAB criteria (s.d.=2.26; range=0–7). A total of 621 (45%) participants (468 males and 153 females) met criterion A for ASPD, defined by endorsement of three or more of the seven criteria. Males had higher AAB criterion counts than females (male mean=3.45, female mean=1.53; *P*<0.0001). Age was negatively associated with AAB criterion counts (*r*=−0.29, *P*<0.0001), indicating that older participants endorsed fewer lifetime AAB criteria. As expected, individuals classified as alcohol-dependent cases in the sample had higher AAB criterion counts (mean 3.8, s.d.=2.0) than those without alcohol dependence (mean 0.67, s.d.=0.96, *P*<0.0001).

**Figure 1:**
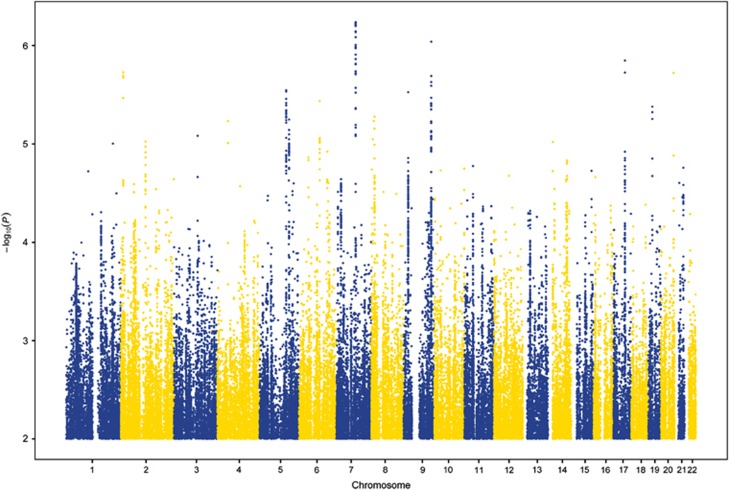
Association results from the genome-wide association study of adult antisocial behavior. On the *x* axis are single-nucleotide polymorphism (SNP) positions for chromosomes 1–22. On the *y* axis are negative logarithms (base 10) of the *P*-values for each SNP, whereby higher values indicate smaller *P*-values.

According to GCTA, the heritability of AAB was moderate, but the estimate did not reach statistical significance (*h*^2^=0.25, s.e.=0.20, *P*=0.09). The GWAS results are summarized in a Manhattan plot in Figure 1 and the quantile–quantile plot is shown in Supplementary Figure S1. The genomic inflation factor was acceptable, *λ*=1.004, suggesting that technical issues and population stratification did not inflate the results. No single SNP met the strict genome-wide significance threshold (*P*⩽5 × 10^−8^). The most highly associated SNP was rs4728702 (*P*=5.8 × 10^−7^), located in the protein-coding gene adenosine triphosphate-binding cassette, sub-family B (MDR/TAP), member 1 (*ABCB1*) on chromosome 7. The top SNP results (*P*⩽5 × 10^−6^) from this analysis are summarized in Table 1.

**Figure 2:**
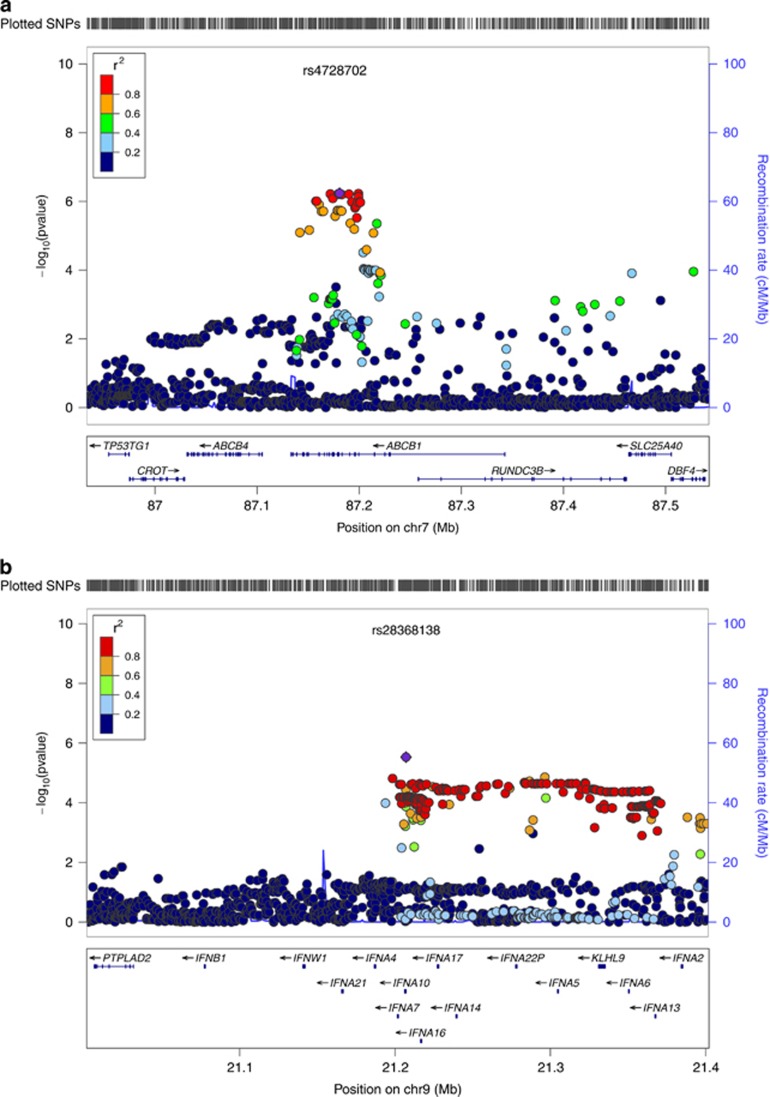

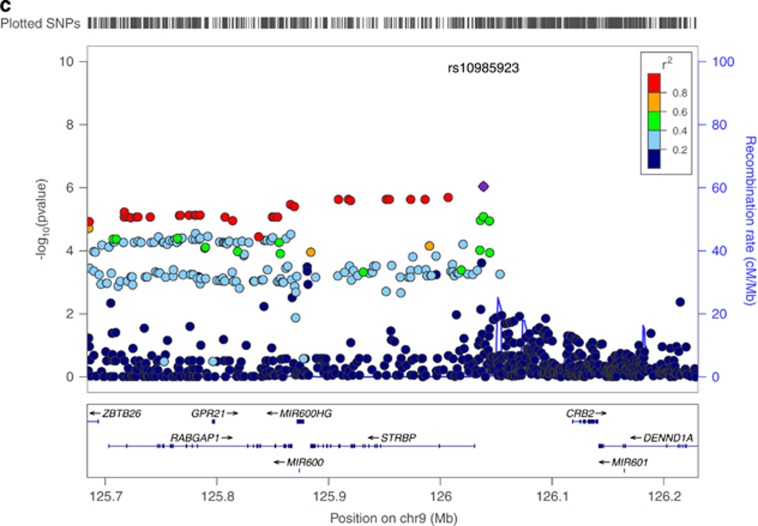
(**a**) Association results from the genome-wide association study (GWAS) of adult antisocial behavior (AAB) for a 200-kb region surrounding rs4728702 in *ABCB1* on chromosome 7. (**b**) Association results from the GWAS of AAB for a 200-kb region surrounding rs28368138 on chromosome 9p. Four genes for type I interferon (*IFNA7*, *IFNA10*, *IFNA16* and *IFNA17*) depicted here reached nominal significance (*P*-value threshold of less than 1 × 10^−4^) in gene-based tests. (**c**) Association results from the GWAS of AAB for a 200-kb region surrounding rs10985923 on chromosome 9q. Five genes (*STRBP*, *MIR600*, *RABGAP1*, *MIR600HG* and *ZBTB26*) depicted here reached nominal significance (*P*-value threshold of less than 1 × 10^−4^) in gene-based tests. All plots were constructed using LocusZoom.^35^ Shown on the *x* axis of all figures are position numbers, and on the *y* axis are the negative logarithms (base 10) of the *P*-values for each single-nucleotide polymorphism (SNP), whereby higher values indicate smaller *P*-values, and local estimates of recombination rates. SNPs are color coded to visualize the haplotype structure in this region, with orange and red coloring indicating SNPs higher in linkage disequilibrium (LD) and blue and green indicating SNPs lower in LD.

In our gene-based test, *ABCB1* met the threshold for genome-wide significance (*q*-value=0.03). This gene was robustly expressed in brain tissue (maximum expression value across nine brain regions=210). The regional association plot for this gene is shown in Figure 2a. The 17 genes from this analysis meeting a *P*-value threshold of less than 1 × 10^−4^ are listed in Table 2, along with their expression values. Two clusters of genes on chromosome 9 appear in this list. As shown in Figures 2b and c, four of these genes are genes for type I interferon (*IFNA7*, *IFNA10*, *IFNA16* and *IFNA17*) on chromosome 9p, and five others (*STRBP*, *MIR600*, *RABGAP1*, *MIR600HG* and *ZBTB26*) appear in a cluster on chromosome 9q.

Enrichment analyses indicated that the genes meeting more stringent significance criteria were more likely to fall into seven known canonical pathways and gene ontologies that are summarized in Table 3. Most of these gene sets are immune related. Many of the same genes were represented across multiple gene sets, and thus these sets are not independent.

### Robustness check and pleiotropy analyses

In our robustness check, the association between *ABCB1* variation and AAB was attenuated after controlling for alcohol dependence, but did not disappear (*P*=1.3 × 10^−2^). Gene-based tests do not have corresponding effect sizes, but the beta coefficient for our most significantly associated SNP (rs4728702) was reduced from −0.36 to −0.17. The sample averages for the four drug classes analyzed as part of our pleiotropy analyses are shown in Supplementary Table S1. The results from a series of gene-based tests indicated that *ABCB1* variation was associated with alcohol dependence criteria (*P*=7.1 × 10^-5^) and cocaine dependence criteria (*P*=0.01), but not cannabis dependence criteria (*P*=0.19) or opioid dependence criteria (*P*=0.50).

### Replication attempt

The sample average for AAB in the COGA fGWAS sample was 1.76 criteria (s.d.=1.76; range=0–7), which was significantly lower than in the case–control data set (unpaired *t*-test: *t*(3137)=11.21, *P*<0.01). A total of 498 (28%) participants (330 males and 168 females) met criterion A for ASPD. There was no evidence for replication of *ABCB1* with AAB in the fGWAS sample (*P*=0.88), nor did any of the other genes or gene sets from Tables 2 and 3 replicate at *P*<0.05.

## Discussion

Historically, molecular genetic research on AAB has been limited to the examination of a small number of candidate genes with purported biological relevance; only recently have researchers begun to conduct atheoretical genome-wide scans for this phenotype.^13, 15, 16^ In our genome-wide investigation, we found that autosomal SNPs accounted for ~25% of the variation in a dimensional measure of AAB. Although this estimate was not statistically significant (*P*=0.09), which is likely attributable to our modest sample size, it maps nicely to meta-analytic findings that additive genetic influences account for 32% of the variation in antisocial behavior.^7^ Our finding also maps to recent GCTA analyses in a community-based sample, where it was found that common genetic variation accounted for 26% (*P*=0.002) of the variation in a behavioral disinhibition phenotype.^16^

No SNP reached genome-wide significance in our GWAS of AAB. Our most associated SNP, rs4728702, was located in *ABCB1* on chromosome 7. In our gene-based tests, *ABCB1* was significant at the genome-wide level; however, we did not find an association for this gene in our replication sample. In expression analyses, we also found that *ABCB1* is robustly expressed in human brain. This provides some biologically plausible evidence that *ABCB1* variation could be associated with behavioral outcomes. *ABCB1* codes for a member of the adenosine triphosphate-binding cassette transporters, ABCB1 or P-glycoprotein, which transport molecules across cellular membranes and also across the blood–brain barrier. *ABCB1* is considered a pharmacogenetic candidate gene in view of ABCB1 transporters' ability to change drug pharmacokinetics.

Variation in *ABCB1* has been previously associated with a number of psychiatric phenotypes, including opioid^36^ and cannabis^37^ dependence, as well as with treatment outcomes for depression^38^ and addiction.^39^ The related rodent gene, *Abcb1a*, is differentially expressed in three brain regions (accumbens shell, central amygdala and ventral tegmental area) of alcohol-preferring animals compared with non-preferring animals.^40, 41^ Furthermore, ethanol exposure changes *ABCB1* expression. An *in vitro* study of human intestinal cells found that ethanol exposure increased messenger RNA *ABCB1* expression level, and that these increases were maintained even after a week of ethanol withdrawal.^42^ Similarly, *ABCB1* expression was increased in lymphoblastoid cell lines following ethanol exposure,^32^ and in rodents, *Abcb1a* expression was increased in the nucleus accumbens of alcohol-preferring rats following alcohol exposure.^43^

Taken as a whole, this pattern suggests that *ABCB1* has pleiotropic effects across a number of externalizing spectrum behaviors/disorders, and that its expression is affected by ethanol exposure. The former is consistent with findings from the twin and molecular genetics literature, demonstrating that common externalizing disorders and behaviors (for example, alcohol dependence, other drug abuse or dependence, AAB and conduct disorder) share genetic influences,^18, 44^ and that this shared genetic factor is highly heritable (*h*^2^=80%).^45^ Supplementary analyses in our own sample were consistent with this hypothesis, and we found evidence that *ABCB1* variation was associated with alcohol and cocaine dependence criterion counts. However, we did not find associations between *ABCB1* and marijuana or opioid dependence criterion counts.

We also found evidence for enrichment (*q-*values⩽0.05) across multiple canonical pathways and gene ontologies including cytokine activity, Jak-STAT signaling pathway, toll-like receptor signaling pathway, antigen processing and presentation, cytokine receptor binding and natural killer cell-mediated cytotoxicity. Although the immediate biological relevance of these categories to AAB is not clear, these enrichment findings include many immune-related pathways and may be best interpreted in light of the associations among AAB and alcohol, cannabis, cocaine and opioid dependence criterion counts in the sample. Immune and inflammatory pathways have been hypothesized to be associated with psychiatric disorders across the internalizing and externalizing spectra.^46^ For example, it is known that alcohol alters cytokine activity,^47^ induces changes in neuroimmune signaling in the brain^48^ and that alcohol dependence is associated with low-grade systemic inflammation.^49^ Likewise, the monocytes of individuals who are cocaine dependent show decreased expression of tumor necrosis factor-α and interleukin-6 proinflammatory cytokines in response to a bacterial ligand relative to controls.^50^ Four of the top genes (based on *P*-values) to emerge in our analysis are genes for type I interferon (*IFNA7, IFNA10, IFNA16 and IFNA17*), which reside in a cluster on chromosome 9p. Previous studies demonstrate that interferon A treatment of hepatitis C patients can induce multiple psychiatric symptoms including depression^51^ and impulsivity.^52^ Although we did not find significant enrichment for these pathways in our replication sample, these results add preliminary evidence to a growing literature that variation in genes in immune-relevant pathways may predispose individuals to AAB and closely related behaviors.

The present study expands upon the initial AAB GWAS by Tielbeek *et al.*^13^ as well as more recently published GWAS of a behavioral disinhibition phenotype^15, 16^ in two important ways. First, we used a case–control sample where the cases met criteria for alcohol dependence. By virtue of the association between alcohol dependence and AAB, and the relatively high rates of individuals meeting clinical cutoffs for criterion A for ASPD in the present sample (63% of males and 24% of females) compared with American population-based prevalence estimates, it is likely that the sample was enriched for genetic variants predisposing individuals toward externalizing spectrum behaviors such as AAB. Previous work indicates that the genetic influences on AAB completely overlap with the genetic influences on alcohol dependence, other drug abuse/dependence and conduct disorder—that is, AAB does not have unique genetic influences above and beyond those shared with these other externalizing disorders.^18^ In view of this, gene identification efforts for AAB are likely to be more successful in more severely affected samples or in samples where participants high in AAB also tend to have comorbid alcohol or substance-use disorders, such as the COGA sample. In contrast, for example, only 6% of the participants in the Tielbeek *et al.*^13^ community-based sample met their non-diagnostic AAB case criteria. This sample may also have had low rates of comorbid alcohol and other drug diagnoses, limiting the ability to find genome-wide significant effects. Second, we used a dimensional measure of AAB, which is more powerful than a binary diagnostic variable, and better represents the underlying dimensional structure of AAB.^53^ These differences may explain, in part, why we were able to detect a significant genetic association in the present sample.

Our study should be interpreted in the context of several limitations. First, our sample size was relatively small. Second, because the COGA case–control alcohol dependence sample is highly affected by AAB, the findings emerging from our study may not generalize to lower-risk populations or other types of high-risk populations. Our null replication attempt may be attributable, in part, to the replication sample being relatively less affected than the discovery sample. There are other instances where genetic associations for externalizing behaviors have replicated within highly affected samples, but not less-affected samples. For example, *GABRA2* is associated with alcohol dependence in samples where alcohol-dependent cases came from clinically recruited samples and families densely affected by alcoholism,^54, 55, 56^ but not community-based samples.^57^ A sample recruited for this purpose is likely to be enriched for genetic variation that predisposes individuals to a range of externalizing behavior problems, including AAB;^18^ however, whether our findings generalize to other populations at high risk for AAB (for example, incarcerated inmates^58^) is unknown. Third, because we limited the current analyses to European-Americans, our results may not generalize to other racial and ethnic groups.

Fourth, similar to all psychiatric outcomes, antisocial behavior has a developmental component, and evidence from the twin literature suggests that there are genetic influences on adolescent and adult antisocial behavior that are distinct from genetic influences on child antisocial behavior.^4^ The degree to which the genetic associations documented here for AAB are also associated with child or adolescent antisocial behavior is not clear. The results from this study provide an empirical starting point for subsequent developmental analyses to examine these questions. Fifth, there are likely to be aspects of the environment that moderate genetic influences on AAB that we did not explicitly examine here but that may be valuable to pursue in subsequent studies. Finally, our genome-wide association approach examined only common genetic variation. There is suggestive evidence that rare nonsynonymous exonic SNPs account for 14% (*P*=0.05) of the variance in a behavioral disinhibition phenotype.^16^ As rare variant-genotyping arrays and whole-genome sequencing become more widely available and cost effective, our understanding of the genetics of AAB will improve.

In summary, our goal in this study was to take an atheoretical approach to investigate the molecular genetic basis of AAB in a high-risk sample. The heritability of AAB was 25%, although this estimate did not differ significantly from zero. No SNP reached strict genome-wide significance, but gene-based tests identified an association between *ABCB1* and AAB. Expression analyses further indicated that *ABCB1* is robustly expressed in the brain, providing some evidence that variation in this gene could be related to a behavioral outcome. Previously documented associations between variants in *ABCB1* and other drugs of abuse suggest that *ABCB1* may confer general risk across a range of externalizing behaviors, rather than risk that is unique to AAB. This was consistent with *post hoc* analyses in our sample, where we found that variation in *ABCB1* was associated with DSM-IV alcohol and cocaine dependence criteria. These pieces of evidence suggest that *ABCB1* may be a gene of interest for further study. We also found enrichment of several immune-related canonical pathways and gene ontologies, which is consistent with previous suggestions that immune and inflammatory pathways are associated with externalizing spectrum behaviors. As a whole, our study goes beyond the candidate gene approach typically taken in studies of AAB, and implicates a gene and gene sets for which there is convergent evidence from other lines of research. These findings, although novel and promising, would benefit from direct replication.

## Figures and Tables

**Table 1:** Top SNPs (*P*<5 × 10^-6^) from a GWAS of adult antisocial behavior

*SNP*	*CHR*	*POS*	*EFF*	*NEFF*	*AF*	*BETA*	P
rs4728702	7	87180678	A	T	0.43	−0.36	5.77E−07
rs1016793	7	87199182	G	A	0.59	0.37	5.91E−07
rs1128503	7	87179601	A	G	0.43	−0.36	5.97E−07
rs4728700	7	87171659	T	C	0.45	−0.36	6.08E−07
rs868755	7	87189930	T	G	0.41	−0.37	6.15E−07
rs10276036	7	87180198	C	T	0.43	−0.36	6.22E−07
rs3789244	7	87181849	G	T	0.43	−0.36	6.22E−07
rs2235026	7	87182882	T	C	0.43	−0.36	6.32E−07
rs2235021	7	87199264	C	A	0.57	0.36	7.15E−07
rs2235020	7	87199265	T	A	0.57	0.36	7.30E−07
rs2235046	7	87174066	T	C	0.45	−0.35	8.20E−07
rs10985923	9	126038684	G	T	0.93	0.71	9.10E−07
rs6959435	7	87158185	G	T	0.43	−0.36	9.82E−07
rs2373586	7	87157583	A	C	0.43	−0.36	9.85E−07
rs62817660	7	87200529	G	A	0.57	0.35	1.03E−06
rs12539098	7	87200639	T	C	0.57	0.35	1.04E−06
rs11975994	7	87192731	G	A	0.43	−0.35	1.05E−06
rs1016794	7	87198459	G	A	0.57	0.35	1.05E−06
rs2520464	7	87201086	C	T	0.57	0.35	1.06E−06
rs2032582	7	87160618	A	C	0.45	−0.35	1.23E−06
rs1202167	7	87197059	C	T	0.57	0.35	1.37E−06
rs6504898	17	52427312	G	T	0.56	−0.36	1.42E−06
rs1202168	7	87195962	G	A	0.57	0.35	1.53E−06
rs1202169	7	87195850	T	C	0.57	0.35	1.55E−06
rs2235013	7	87178626	C	T	0.51	−0.34	1.83E−06
rs2235033	7	87179143	A	G	0.51	−0.34	1.83E−06
rs12704364	7	87181175	C	T	0.51	−0.34	1.83E−06
rs6961665	7	87181418	C	A	0.51	−0.34	1.83E−06
rs58738000	2	11704426	C	G	0.92	−0.66	1.87E−06
rs6504902	17	52433609	T	C	0.56	−0.35	1.88E−06
rs4810138	20	57144231	G	C	0.33	0.38	1.90E−06
rs2235027	7	87182779	G	T	0.51	−0.34	1.91E−06
rs10234411	7	87164892	T	A	0.45	−0.34	1.94E−06
rs4148738	7	87163049	C	T	0.45	−0.34	1.96E−06
rs68152859	2	11699747	G	A	0.92	−0.67	2.02E−06
rs10985911	9	126007069	T	C	0.93	0.68	2.04E−06
rs5020877	2	11694370	A	G	0.92	−0.67	2.12E−06
rs142285425	9	125951669	A	G	0.93	0.67	2.34E−06
rs10985900	9	125954349	T	C	0.93	0.67	2.34E−06
rs7870519	9	125917716	A	T	0.93	0.67	2.34E−06
rs62579000	9	125973531	C	T	0.93	0.67	2.34E−06
rs10985892	9	125908794	T	C	0.93	0.67	2.35E−06
rs10985908	9	125986335	C	T	0.93	0.67	2.35E−06
rs62578996	9	125919594	G	A	0.93	0.66	2.56E−06
rs10808072	7	87176463	A	G	0.51	−0.33	2.69E−06
rs55980995	5	121875565	A	G	0.88	−0.52	2.84E−06
rs6893509	5	121860590	T	G	0.89	−0.55	2.92E−06
rs28368138	9	21207334	C	G	0.95	−0.82	2.97E−06
rs1202165	7	87197594	G	A	0.59	0.35	3.00E−06
rs12002466	9	125865899	T	C	0.93	0.65	3.40E−06
rs180996880	2	11693084	C	A	0.92	−0.65	3.41E−06
rs74602468	5	121863973	C	T	0.90	−0.56	3.48E−06
rs67242082	6	94334202	A	G	0.86	−0.48	3.66E−06
rs76585835	5	121867413	G	A	0.90	−0.56	3.83E−06
rs41277128	9	125610975	G	A	0.93	0.66	3.85E−06
rs7033878	9	125618080	T	G	0.93	0.66	3.88E−06
rs62580884	9	125619738	C	A	0.93	0.66	3.88E−06
rs10985798	9	125624520	G	C	0.93	0.66	3.88E−06
rs62580922	9	125631594	T	C	0.93	0.66	3.88E−06
rs10985807	9	125652376	C	T	0.93	0.66	3.88E−06
rs78303085	9	125869322	T	A	0.93	0.66	3.96E−06
rs62578960	9	125869287	G	A	0.93	0.66	3.97E−06
rs80233205	5	121871869	G	A	0.90	−0.56	4.03E−06
rs55919124	5	121880314	T	A	0.90	−0.56	4.05E−06
rs62580923	9	125635317	A	T	0.93	0.65	4.13E−06
rs6413435	19	18497137	G	A	0.83	−0.44	4.18E−06
rs6948766	7	87191246	G	A	0.52	−0.33	4.32E−06
rs11763872	7	87217215	T	C	0.49	0.33	4.40E−06
rs7034822	9	125621808	G	A	0.93	0.65	4.46E−06
rs12378760	9	125661283	T	C	0.93	0.65	4.46E−06
rs17149293	5	121864140	G	T	0.90	−0.55	4.53E−06
rs76809806	5	121873792	G	A	0.90	−0.55	4.66E−06
rs12979706	19	18495424	G	A	0.83	−0.44	4.75E−06
rs12514901	5	121878119	T	G	0.89	−0.54	4.84E−06
rs78479583	5	121871427	G	A	0.90	−0.55	4.96E−06

Abbreviations: AF, allele frequency of the effect allele; BETA, regression coefficient; CHR, chromosome; EFF, effect allele; GWAS, genome-wide association study; NEFF, non-effect allele; POS, position (in base pairs); SNP, single-nucleotide polymorphism.

**Table 2:** Top genes associated with adult antisocial behavior

*Gene name*	*Chromosome*	*Gene* P*-value*	*Gene* q*-value*	*Brain exp*
*ABCB1*	7	1.42E−06	0.033	210
*CD46*	1	1.28E−05	0.297	451
*ABCB5*	7	2.06E−05	0.476	—
*IFNA7*	9	2.34E−05	0.542	—
*MIR600*	9	2.53E−05	0.584	NA
*MIR600HG*	9	2.53E−05	0.584	NA
*STRBP*	9	3.55E−05	0.821	359
*GREB1*	2	3.85E−05	0.891	163
*RC3H2*	9	3.88E−05	0.897	498
*ZBTB6*	9	4.28E−05	0.989	190
*IFNA17*	9	4.55E−05	1.000	—
*RABGAP1*	9	7.97E−05	1.000	1228
*NKAIN3*	8	8.04E−05	1.000	281
*OLIG2*	21	8.11E−05	1.000	155
*ZBTB26*	9	8.24E−05	1.000	153
*IFNA16*	9	8.52E−05	1.000	—
*IFNA10*	9	9.68E−05	1.000	—

Brain exp. is the maximum of average expression for nine brain regions; ‘—' is below background; NA indicates measurement not available because the gene was not present on array.

Notes: Gene *P*-value and gene *q*-value refer to the uncorrected *P*-values and the *q*-values based on a Benjamini and Hochberg false discovery rate for the gene-based tests. A gene was presumed to be expressed in the brain if the maximum expression level across the nine regions was higher than 16.0 because this is above the background signal for the arrays. Expression ranges from 5–20,140; median=108; 90th percentile=484. For comparison, the neurotransmitter receptor gene *HTR1B* (serotonin 1D beta receptor) that is normally expressed in the brain has a maximum expression of 182.

**Table 3:** Canonical pathways and gene ontologies

*Gene set name*	*Gene set* q*-value*
Cytokine activity	<0.0001
Jak-STAT signaling pathway	<0.0001
Toll-like receptor signaling pathway	0.0005
Antigen processing and presentation	0.0008
Hematopoietin interferon class D200 domain cytokine receptor binding	0.002
Natural killer cell-mediated cytotoxicity	0.005
Tryptophan metabolism	0.05
