# Supplementary material for: Genome-wide association data suggest ABCB1 and immune-related gene sets may be involved in adult antisocial behavior
Source: Transl Psychiatry. 2015 Apr 28;5(4):e558–. doi: 10.1038/tp.2015.36 (PMC4462601; doi:10.1038/tp.2015.36)

**Supplementary Information**

Table S1. COGA case-control sample descriptive statistics for major drug classes and their association with adult antisocial behavior (AAB).

| Drug class | Mean | Standard deviation | % of sample meeting clinical cutoff | Correlation between AAB and drug class |
| --- | --- | --- | --- | --- |
| Alcohol dependence | 3.40 | 2.90 | 60.46% | 0.71 |
| Marijuana dependence | 1.13 | 1.95 | 20.42% | 0.53 |
| Cocaine dependence | 1.33 | 2.49 | 21.75% | 0.55 |
| Opioid  dependence | 0.45 | 1.52 | 7.37% | 0.32 |
| | *Notes.* The mean and standard deviations reported are for the continuous criterion counts for each disorder according to DSM-IV. The correlations reported are for continuous criterion counts for AAB and each drug class. All correlation *p*-values < 0.0001. | | --- | | | | | |

Figure S1. Quantile-quantile plot from the GWAS of AAB, which plots the observed –log_10_(p) values for each SNP in the analysis (ordered from smallest to largest) against the expected log_10_(p) values under a uniform distribution of p-values.


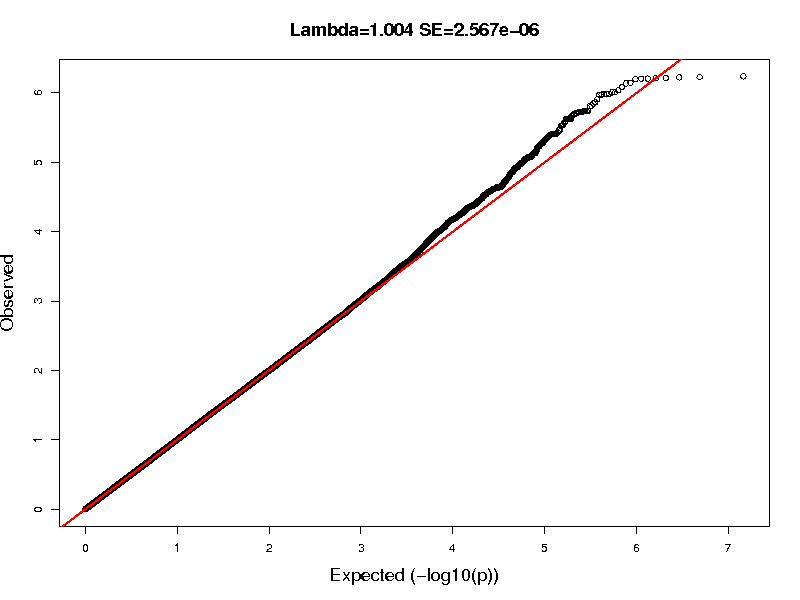

Supplement: Supplementary Information [file tp201536x1.doc]
